# Outer Membrane Proteome Analysis of Indian Strain of *Pasteurella multocida* Serotype B:2 by MALDI-TOF/MS Analysis

**DOI:** 10.1155/2014/617034

**Published:** 2014-12-22

**Authors:** A. Prasannavadhana, Santosh Kumar, Prasad Thomas, Laxmi Narayan Sarangi, Santosh Kumar Gupta, Adyasha Priyadarshini, Viswas Konasagara Nagaleekar, Vijendra Pal Singh

**Affiliations:** ^1^Division of Veterinary Bacteriology and Mycology, Indian Veterinary Research Institute, Izatnagar, Bareilly, Uttar Pradesh 243122, India; ^2^Institute of Bacterial Infections and Zoonoses, Friedrich Loeffler Institut, Namburger Straße 96 a, 07743 Jena, Germany; ^3^Regional Medical Research Centre, Bhubaneswar, Odisha 751023, India

## Abstract

Identification of outer membrane proteins (OMPs) is important to understand the bacteria structure and function, host-pathogen interaction, development of novel vaccine candidates, and diagnostic antigens. But till now the key antigens of *P. multocida* B:2 isolate causing haemorrhagic septicaemia (HS) in animals are not clearly defined. In this study, P52 strain of *P. multocida* serotype B:2 was grown *in vitro* under iron-rich and iron-limited condition. The OMPs were extracted by sarkosyl method followed by SDS-PAGE and the proteins were identified by MALDI-TOF/MS analysis. In total, 22 proteins were identified, of which 7 were observed exclusively under iron-limited condition. Most of the high molecular weight proteins (TbpA, HgbA, HgbB, HasR, IroA, and HemR) identified in this study were involved in iron acquisition. Some hypothetical proteins (HP-KCU-10206, HP and AAUPMB 08244, HP AAUPMB 21592, HP AAUPMB 19766, AAUPMB 11295) were observed for the first time in this study which could be unique to serotype B:2. Further functional *in vivo* study of the proteins identified are required to explore the utility of these proteins in developing diagnostics and vaccine against HS.

## 1. Introduction

Haemorrhagic septicaemia (HS) is an important bacterial disease causing high mortality in cattle and buffaloes. The outbreak of the disease is seen frequently all over India and is responsible for approximately 50–60% of mortality in bovines and other species of animals causing huge economic losses [1]. The causative organism* Pasteurella multocida* belonging to family Pasteurellaceae is grouped into five serogroups A, B, D, E, and F, based on their capsular typing and 16 serotypes based on somatic typing [2, 3]. In India, HS is mostly caused by serotype B:2.

Outer membrane proteins (OMPs) are important virulence factors involved in colonization, invasion, and pathogenesis and many of them have been found to provide protective immunity against* P. multocida* infection [4–6]. Thus, identification of OMPs is critical to understand the bacterial structure and function, host-pathogen interactions, to identify the protective antigens and to develop novel diagnostics [7]. It is important to have thorough knowledge of the outer membrane proteome of* P. multocida* which will help in identification of potential virulence factors, diagnostic antigens, drug targets, and vaccine candidates. Although various workers have used different methods to study the OMPs, proteomic studies by using mass spectrometers (LC MS/MS, MALDI-TOF-MS) combined with bioinformatic tools (protein prediction algorithms/software) have been found promising.

The key antigens of* P. multocida* B:2 that evoke protective immunity against HS in cattle have still not been well defined, but its OMPs have been found as protective antigens [6, 8, 9]. Boyce et al. [5] have studied the OMPs of* P. multocida* during infection of the natural host in chickens and by subjecting sarcosine-insoluble membrane fractions to 2-DE and 1-DE followed by MALDI-TOF/MS and nano-LC MS/MS analysis and have identified 35 proteins. A putative iron-regulated porin (Pm0803) was also identified which was highly upregulated under both* in vivo* and iron-limited growth conditions. Wheeler [10] studied the comparative analysis of the OM proteome of eight* P. multocida* isolates recovered from different hosts and observed that HgbA and TbpA were not predicted from the avian Pm70 genome but were expressed by bovine and ovine isolates, providing evidence of the importance of these OMPs to the broad host range of* P. multocida*.

The previous studies carried out on outer membrane proteomics of* Pasteurella multocida* serotype B:2 were based on sodium dodecyl sulphate-polyacrylamide gel electrophoresis (SDS-PAGE) analysis and have identified proteins based on molecular weights (m.w.). As different OMPs show molecular weights variation, identification of proteins solely based on molecular weights could be misleading. These shortcomings can be overcome by MALDI-TOF analysis where proteins are identified with precision. Thus, in this study, this technique was extended to serotype B:2 isolate.

## 2. Materials and Methods

### 2.1. Bacterial Strain and Antisera

P52 strain of* P. multocida* serotype B:2 was used in the present study. This strain was isolated from buffalo and is currently used as vaccine strain for production of HS vaccine in India. The lyophilized cultures were revived in brain heart infusion (BHI) broth and incubated overnight at 37°C. The purity and identity of the cultures were tested by morphological, cultural, and biochemical examinations as per standard procedures [11]. Molecular characterization of* P. multocida* was carried out by PM-PCR, multiplex PCR, and HS-B PCR assays [12, 13].

For western blotting, different types of serum, namely, apparently healthy animal sera, hyperimmune sera, experimentally infected animal sera, and field sera against* P. multocida* serotype B:2, maintained in the division of Bacteriology and Mycology, Indian Veterinary Research Institute, were used.

### 2.2. Optimization of Iron-Limited Culture Conditions

To create iron-limited culture condition the bacterial cultures were grown in BHI broth containing the iron-chelating agent 2,2′-dipyridyl (Sigma Aldrich, USA). The concentration of dipyridyl capable of inducing observable expression of iron-uptake OMPs without completely inhibiting growth was determined by inoculating the colonies in 5 mL BHI broth containing 0, 50, 100, 150, 200, 250, 300, 350, and 400 *μ*M of 2,2′-dipyridyl.

### 2.3. Growth under Iron-Rich and Iron-Limited Conditions

Loop full of* P. multocida* colonies grown in blood agar was inoculated in 5 mL of BHI broth and incubated overnight at 37°C in orbital shaker incubator at 120 rpm. For batch culture, 400 mL of prewarmed BHI broth was inoculated with 400 *μ*L (1%) of overnight culture. In case of iron-regulated condition the appropriate concentration of 200 *μ*M of sterile 2,2′-dipyridyl was also added and both cultures were incubated at 37°C with shaking at 120 rpm for 6–8 h until cultures reach mid-log phase equivalent to an OD of ~1.0 at 600 nm.

### 2.4. Outer Membrane Protein Extraction by Sarkosyl

The OMP fractions of* P. multocida* serotype B:2 were prepared as per method described by Davies et al. [14] and Wheeler [10] with slight modifications. The overnight grown cultures were transferred to 250 mL centrifuge bottles and centrifuged at 10,000 g at 4°C for 30 min to pellet the cells. The supernatant was discarded and the bacterial pellet was resuspended in 50 mL ice cold 20 mM Tris HCl (pH 7.2). The suspension was again centrifuged at 10,000 g for 30 min at 4°C. After discarding the supernatant, the pellet was resuspended again in 8.0 mL of ice cold 20 mM Tris HCl (pH 7.2). The suspended cells were lysed by sonication in ice using a Soniprep 150 sonicator (MSE UK Ltd.). The sequence followed for sonication was 12 *μ*m for 45 sec followed by 45 sec gap. The cycle was repeated for 10 times. The lysate was centrifuged at 10,000 g in round bottom tubes for 30 min and the supernatant was collected. The collected supernatant was carefully transferred to 10 mL ultracentrifuge tubes and centrifugation was done by using himac CP 80b (Hitachi, Japan) at 50,000 g for 1 h at 4°C. The supernatant was discarded and the gelatinous pellets were suspended in 8 mL 0.5% sodium N-lauroylsarcosine (Sigma Aldrich, USA) using long form of Pasteur pipettes. The sarkosyl insoluble outer membrane fraction was pelleted by centrifugation at 50,000 g for 1 h at 4°C. The pellet was resuspended in 10 mL of 20 mM Tris-HCl (pH 7.2), and the suspension was centrifuged again at 50,000 g for 1 h at 4°C. The small amount of outer membrane pellet obtained was resuspended in small volume (less than two mL of 20 mM Tris-Hcl, pH 7.2) and was stored at −20°C.

The concentration of OMP was determined by modified Lowry assay as described by Markwell et al. [15] and Wheeler [10].

### 2.5. SDS-PAGE and Western Blotting

SDS-PAGE analysis was carried out as per the protocol described by Laemmli [16] with slight modifications suggested by Wheeler [10]. The OMPs separated in SDS-PAGE were blotted electrophoretically onto nitrocellulose membrane (NCM) using semidry western blotting apparatus (ATTO, Japan) following the protocol described by Colligan et al. [17] with minor modifications. Gel was kept in transfer buffer for 5–10 min. After transfer to the NCM, the membrane was placed in blocking buffer and incubated at 4°C overnight followed by washing twice with TBS-T for 10 min each. Different type antiserums present in the laboratory were diluted in the blocking buffer (1 : 500) and were used as primary antibody. Membrane was incubated for 1 h at room temperature with constant agitation. The membrane was washed four times with TBS-T for 10 min each and then incubated at room temperature in rabbit antibovine HRPO IgG conjugate (Sigma Aldrich, USA) for 1 h with constant agitation. The membrane was washed four times in TBS-T and the blots were developed by immersing it in the chromogenic visualization solution for 5–10 min. The reaction was terminated by washing the membrane with distilled water and then air-dried and photographed.

### 2.6. MALDI-TOF/MS

The destained gel was transferred to a clean, sterile, and transparent plastic plate by following sterile precautions. The selected gel bands were cut carefully and each gel band was transferred to sterile Eppendorf tubes and rinsed with autoclaved triple distilled water by gentle pipetting. The distilled water was drained completely and the tubes containing gel bands were sealed with Parafilm, labelled, and dispatched for MALDI-TOF/MS (Indian Institute of Science, Bangalore, India). In total, 16 protein bands were selected for MALDI-TOF/MS analysis including 8 protein bands (N1–N8) cut from OMPs grown in iron-rich condition and 8 (IR1–IR8) from OMPs grown in iron regulating condition.

### 2.7. NCBI-BLASTP Analysis

The peptide mass fingerprints (PMF) obtained from MALDI-TOF/MS analysis were further analyzed by BLASTP (NCBI, USA) to check the identity of the results [18]. The database search was restricted to the phylum Proteobacteria and allowed for a maximum of one missed cleavage, modification by carbamidomethylation, variable modification of methionine residues by oxidation, and a positive peptide charge of 1.

## 3. Results

### 3.1. Optimization of Iron-Limited Culture Conditions

The different concentrations of 2,2′-dipyridyl were used to optimize the concentration for batch culture and the appropriate concentration of 2,2′-dipyridyl which induced the maximum observable growth was found to be 200 *μ*M.

### 3.2. Determination of OMP Concentration

The concentration of OMP obtained by ultracentrifugation was determined using modified Lowry assay and the concentration of OMP in iron-rich culture was found to be 4.30 mg/mL while the concentration in iron-limited culture was 5.2 mg/mL. The concentration was adjusted to 2 mg/mL by adding Tris-HCl buffer pH 7.2.

### 3.3. SDS-PAGE for OMPs

**Figure 1:**
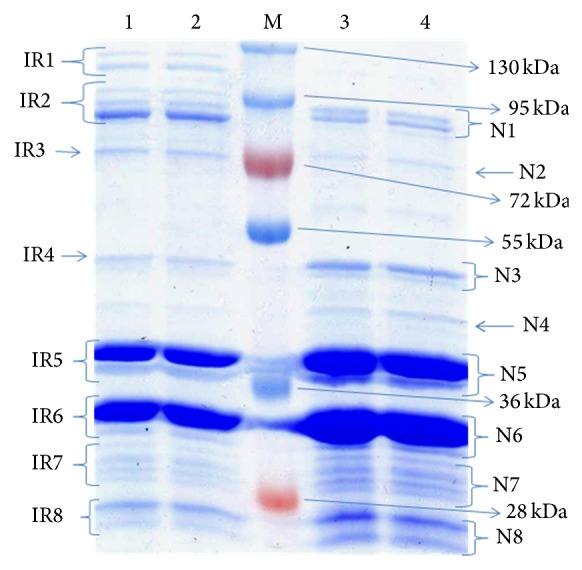
Coomassie blue-stained SDS-PAGE showing outer membrane protein profile of* Pasteurella multocida* serotype B:2 (P52) strain cultured in iron-rich or iron-limited media. Lanes 1, 2: OMPs growing in iron limiting condition, lane M: prestained protein ladder, and lanes 3, 4: OMPs growing in iron-rich condition. IR: bacteria grown under iron-regulated condition; N: bacteria grown under normal condition (iron-rich condition). IR-1 to IR-8 and N1 to N8 represent the portion of the gel slice cut and sent for MALDI-TOF/MS analysis. The proteins identified in the MASCOT analysis are presented in Table 1.

The SDS-PAGE profile of OMPs under iron-rich and iron-limited conditions is depicted in Figure 1. The molecular weights of the polypeptide bands were estimated by comparison with standard molecular weights markers run in parallel. OMP profiles comprised of two major polypeptide bands (38 and 33 kDa) and 14-15 other minor polypeptide bands. The molecular mass of the polypeptide bands ranges from approximately 23 to 92 kDa in iron-rich condition. Three high molecular weights proteins with approximate molecular weights, namely, 127 kDa, 125 kDa, and 110 kDa, were observed exclusively in iron-limited conditions. It was also found that 87 kDa protein was upregulated in iron-limited conditions while the protein bands that were downregulated in iron-limited when compared to normal conditions were found to be 53, 38, 36, 35, 33, and 32 kDa.

### 3.4. MALDI-TOF/MS

In the present study, sixteen protein bands were selected from both preparations (iron-rich and iron limiting condition) for MALDI-TOF/MS analysis. The protein bands were selected based on the combined results of SDS-PAGE and the western blotting and the preference was given to iron-regulated proteins and the immunodominant proteins to identify the novel proteins of* P. multocida*. The OMPs were identified from the peptide mass fingerprinting output by the MASCOT (Matrix Science) sequence matching software with Ludwig NR database, and the results of MALDI were further analyzed by BLASTP. Proteins were identified using the Mascot search engine (Matrix Science). The results of the mass spectrometric analysis showed that more than one protein was identified from respective bands in both iron-rich and iron-limited condition preparations. The identified proteins were shown in Table 1.

In total 22 OMPs were identified by MALDI-TOF/MS analysis which includes TbpA, HgbA, PM0336 (HgbB), HasR, IroA, Oma87 (PM1992), HP-KCU-10206, HP AAUPMB 08244, HP AAUPMB 21592, HP AAUPMB 19766, AAUPMB 11295, HmbR, long-chain fatty acid transport protein, 47 kDa protein, PM1069 (OMP P1 precursor protein), TolB, TolC, HexD, CexD, OmpA, PM786 (Omp34), OmpH (39 kDa protein), and glycerophosphodiester phosphodiesterase. Among these proteins HgbA, IroA, HasR, tolB, hexD, tolC, and cexD were observed only in iron limiting condition whereas glycerophosphodiester phosphodiesterase protein was detected only in case of OMPs grown in iron-rich condition.

### 3.5. Western Blotting

**Figure 2:**
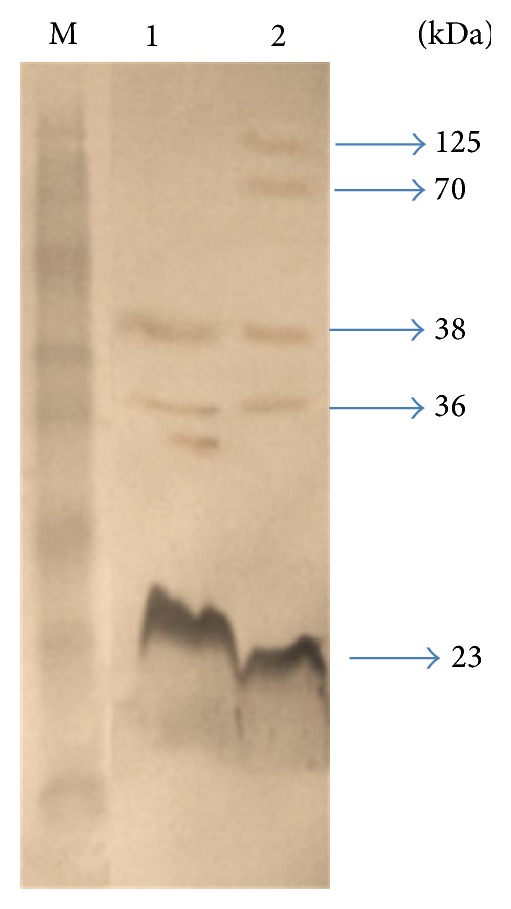
Immunodominant outer membrane proteins of* Pasteurella multocida* B:2 in hyperimmune sera. Lane M: protein marker (170–15 kDa). Lane 1: immunodominant protein in normal condition. Lane 2: immunodominant proteins in iron-limited condition.

**Figure 3:**
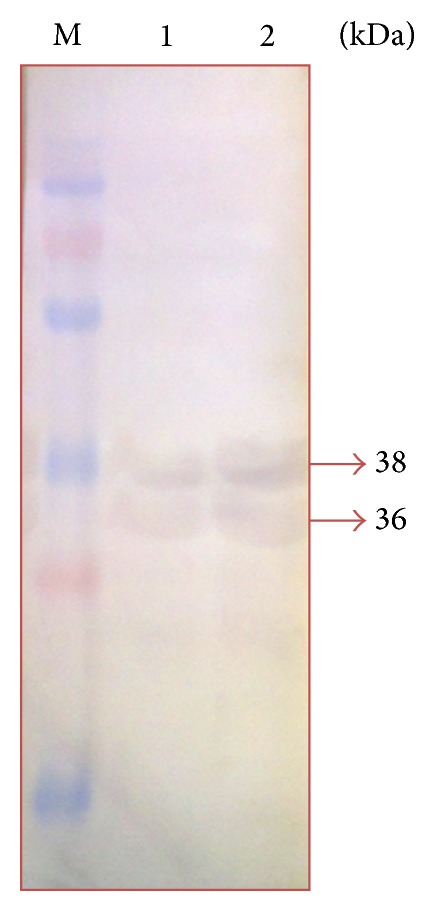
Immunodominant outer membrane proteins of* Pasteurella multocida* B:2 in apparently normal animal sera. Lane M: protein marker (250–15 kDa). Lane 1: immunodominant protein in normal condition. Lane 2: immunodominant proteins in iron-limited condition.

**Figure 4:**
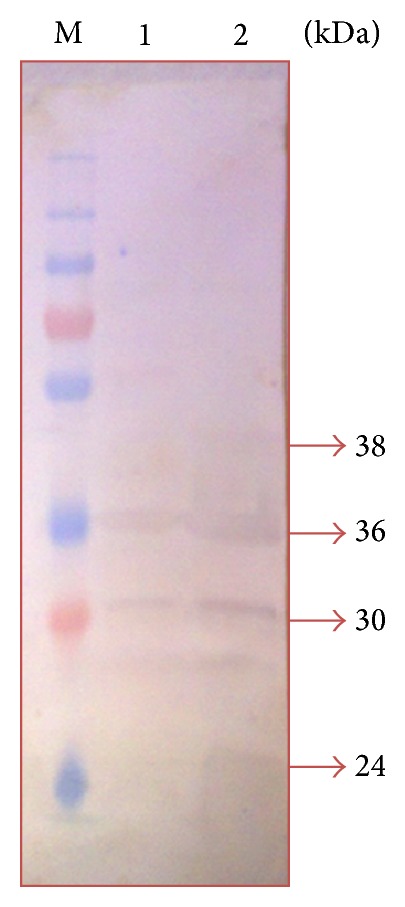
Immunodominant outer membrane proteins of* Pasteurella multocida* B:2 in field sera. Lane M: protein marker (250–10 kDa). Lane 1: immunodominant proteins in iron-limited condition. Lane 2: immunodominant protein in normal condition.

**Figure 5:**
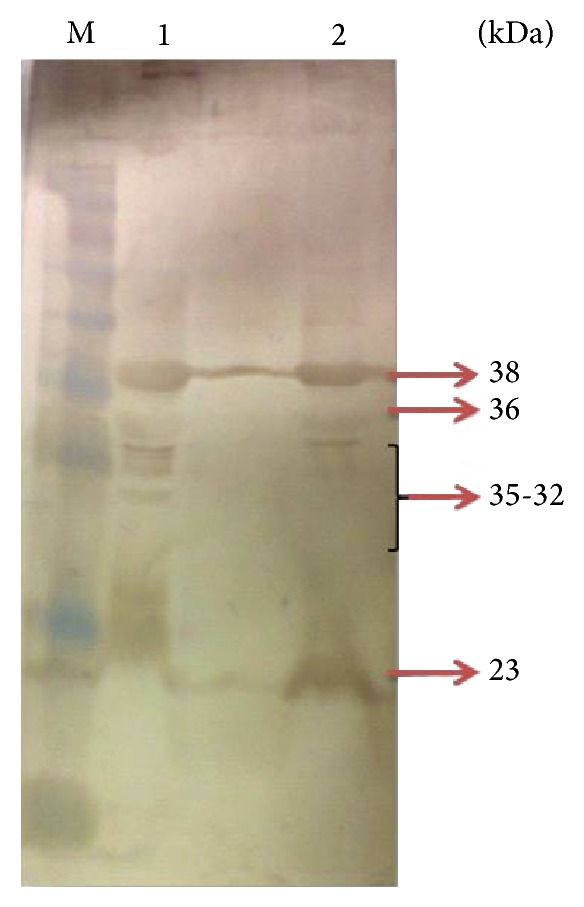
Immunodominant outer membrane proteins of* Pasteurella multocida* B:2 in vaccinated sera. Lane M: protein marker (170–15 kDa). Lane 1: immunodominant protein in normal condition. Lane 2: immunodominant proteins in iron-limited condition.

Western blot analysis showed the presence of immunodominant OMPs of* P. multocida* serotype B:2. It was found that the immunodominant protein bands obtained in the immunoblots differed with the type of antisera used as shown in Figures 2, 3, 4, and 5. A total of five immunodominant proteins, namely, 125, 70, 38, 36, and 23 kDa, were found using hyperimmune sera, four proteins 38, 36, 30, and 24 kDa using field sera, six proteins 38, 36, 35, 33, 32, and 23 kDa using vaccinated animal sera, and two proteins 38, 36 kDa using healthy animal sera. The details of the immunodominant bands are shown in Table 2.

## 4. Discussion

Outer membranes are the important structures and major immunogens of many Gram negative bacteria including* P. multocida* which contribute to virulence of the organisms. The OMPs of* P. multocida* were found to be immunogenic in buffalo calves which indicate that it can be used to develop vaccines against HS [19]. It has also been reported by many workers that the iron-regulated OMPs of* P. multocida* were immunogenic against homologous and heterologous challenges [20–22]. OMPs of* P. multocida* are known to alter their expression according to the host environment [5]. So, to find out the iron-regulated OMPs and to understand the expression pattern of* P. multocida* serotype B:2 under iron-limited condition,* P. multocida* were grown in liquid media containing an iron chelator.

The OMP profile of serotype B:2 of* P. multocida* was typical of Gram negative bacteria. Similar type of SDS-PAGE profile has also been reported by other workers with slight difference in molecular weights [10, 19, 23–26]. Determination of molecular weights solely on basis of molecular marker is erroneous and could have led to such discrepancies. However, the difference in the serotypes subjected to the study and the pressure of the host environment* in vivo* on the expression of the protein can also cause difference in the molecular weights of the proteins identified in SDS-PAGE. Similarly, it has been reported that the same organism at different passage level* in vitro* may also express proteins of different molecular weights [27]. Therefore, expression of proteins solely on the basis of molecular weights could be misleading. In this study, MALDI-TOF/MS analysis was carried out to identify these proteins with precision.

Many bacteria possess more than one iron sequestering system to obtain iron from one source. All Gram negative bacteria including those belonging to family Pasteurellaceae have been shown to express numerous ton B dependent iron binding proteins. In* P. multocida* three types of iron-uptake system have been recognised which can be used to extract iron directly from transferrin, haemoglobin, and haemophores. In this study, a number of proteins involved in iron acquisition were observed. They are mostly high molecular weights proteins like TbpA, HgbA, HgbB, IroA, HasR, and HmbR. Among these, TbpA protein was identified from two different polypeptide bands appearing around 90–95 kDa and 75–80 kDa in SDS-PAGE. These two proteins could be Tbp1 and Tbp2. The size of Tbp1 protein ranges from 90 to 100 kDa and Tbp2 from 65 to 85 kDa [28] and both these proteins can bind to transferrin and have been found in HS causing isolates [29, 30]. The other proteins which were identified in high molecular weights region were Oma87 (PM1992), Alanyl tRNA ligase, and ligand-gated channel proteins. Among these Oma87 is a highly immunogenic protein involved in cross protection. It shares extensive similarity with D15 protective surface antigen of* Haemophilus influenza* [20, 24].

The two most intense bands observed in the SDS-PAGE (33 kDa and 38 kDa) were identified as OmpA and OmpH, respectively, by MASCOT search. The molecular weights of these proteins are known to vary among strains and in SDS-PAGE with heat treatment. In this study, OmpA and OmpH were found at different locations on the SDS-PAGE (23 to 38 kDa protein), the reason of which is unclear. Similar observation has been reported previously [5, 26]. It has been reported that membrane proteins might migrate anomalously in SDS-PAGE and the gel molecular weights may not correspond to the actual molecular weights based on amino acid composition ([31] and reference therein).

Some other proteins identified in the low molecular weight region were TolC, TolB, HexD, CexD, 47 kDa protein, PM1069 (OMP precursor P1 protein), long-chain fatty acid transport protein, membrane proteins belonging to hydrocarbon dehydration family, glycerophosphodiester esterase, Mlt-A interacting mipA protein, and type 4 fimbrial biogenesis protein pilZ. Among these TolC is a key component of both type I secretion system and efflux pump and recent evidence suggests that they are involved in multidrug resistance in bacteria [32, 33]. The 47 kDa protein is an adhesion and its variant molecular weight form has been identified [10].

Few hypothetical proteins (HP-KCU-10206, HP AAUPMB 08244, HP AAUPMB 21592, HP AAUPMB 19766, and AAUPMB 11295) were also identified in this study for the first time. The protein sequences of these hypothetical proteins matched only with the draft genome of* P. multocida* P52 or Anand Buffalo isolate submitted to NCBI. Therefore, these proteins could be the novel proteins present only in Indian isolates. The identity of these proteins and their function will be clearer once the annotation of the whole genome is completed.

A number of OMPs of* P. multocida* serotype B which have been identified in previous proteomics studies and/or by genomic studies (detection by PCR technique followed by cloning and expression of* P. multocida* serotype B:2 P52 strain) were not detected in the present study [10, 34–36]. This could be due to incomplete identification of the OMPs or because of vigorous methods used in sarkosyl extraction technique in which many OMPs are lost. The other possibility is the low level expression of some of these OMPs which could not be detected by MADI-TOF/MS analysis. Therefore, use of more sensitive techniques like nano-LC/MS and 2-DE followed by MALDI-TOF/MS analysis along with use of bioinformatics software could lead to identification of more number of proteins.

The results of western blot analysis for detection of immunodominant OMPs suggested that 23, 24, 36, and 38 kDa proteins were major immunodominant OMPs irrespective of the type of sera used (Figures 2–5). This may be due to the fact that these OMPs are also expressed by avirulent strains of* P. multocida* which is a normal commensal of bovine respiratory tract. Similarly, the low molecular weight proteins, namely, 38 and 36 proteins, which were also detected in healthy animal serum, suggests that these proteins share some common antigen with other bacteria colonizing respiratory tract and they may not be suitable candidates for developing differential diagnostics for* P. multocida* serotype B:2. However, the 125 kDa protein which is only present in iron-limited condition and 70 kDa protein which is common for both iron-limited and iron-rich condition were found to be immunodominant with hyperimmune sera that rose against* P. multocida* serotype B:2 in bovine calves. More or less similar observations have been reported in the western blot analysis by previous workers [8, 19, 24–26, 31, 37]. 32 kDa and 37 kDa protein have been reported to be the most immunogenic proteins of* P. multocida* serotype B isolates. Except for these proteins, some authors also reported 16, 20, 28, 44, 48, 50, 56, 86, and 90 kDa proteins to be immunogenic [8, 19, 24–26, 31, 37]. These differences in the observations could be due to the different serotypes used in the study and/or individual animal factors in eliciting the immune response to these proteins. In addition, some immunodominant proteins may not get detected by antibodies due to denaturation of conformational epitopes in SDS-PAGE [38].

Comparing the results of MALDI-TOF analysis with SDS-PAGE and western blotting it could be speculated that OmpA and OmpH correspond to the 33 kDa and 38 kDa protein and are the most immunogenic proteins of* P. multocida*. Similarly, HgbA and TbpA could be the other proteins (70 kda and 125 kDa) found to be immunogenic in western blot analysis. The polypeptide bands corresponding to 23, 24, 25, 32, 33, 35, 36, and 38 showed homology with OmpA or OmpH in MASCOT search. No other proteins (except HP AAUPMB 08244, HP AAUPMB 21592) were identified in these regions by MASCOT search. The probability of OmpA and OmpH obscuring another protein of similar molecular weight present at very low concentration could not be ignored. The other proteins which have been found to be immunogenic in* P. multocida* serotype B:2 in previous studies are omp16, oma 87, hasR, plpB, and so forth [23, 24, 31, 37]. To confirm the authenticity and to ascertain the importance of these proteins the OMP profiling of isolates recovered from normal (commensal) animals and isolated from HS cases (pathogenic) should be carried out in the future covering all parts of country.

## 5. Conclusion

In this study, 22 proteins of* P. multocida* serotype B:2 grown under iron-rich and iron-limited condition were identified by MALDI-TOF/MS analysis. Of these, iroA, translocation protein TolB, HexD, partial, TolC protein, CexD, MltA-interacting MipA protein, and partial and alanyl-tRNA ligase were exclusively observed in iron-regulated conditions, whereas glycerophosphodiester phosphodiesterase protein is present only in case of OMPs grown in iron-rich condition. These proteins could be novel differentially expressed proteins under iron limitation. Similarly, few hypothetical proteins (HP-KCU-10206, HP AAUPMB 08244, HP AAUPMB 21592, and HP AAUPMB 19766) were observed for the first time in this study which could be unique to serotype B:2. In western blot analysis, using different serum, OmpA, OmpH, 47 kDa protein, HgbA, and TbpA were found to be immunogenic. Due to the limitation of the overall procedure, a number of OMPs could not be detected in this study. Therefore, 2-DE followed by nano-LC/MS along with use of bioinformatics software for prediction of OMPs following whole genome sequencing should be carried out in the future to identify more proteins. Further functional* in vivo* studies are needed to understand the role of the identified proteins in pathogenicity and virulence of the organisms and to explore the utility of these proteins in developing diagnostics and vaccines against haemorrhagic septicaemia.

## Figures and Tables

**Table 1:** Outer membrane proteins of *Pasteurella multocida* serotype B:2 identified by Mascot and BLASTP analysis.

Band ID^a^	MASCOT search homology with species and NCBI accession number	Molecular weight (Da)	Isoelectric point	Sequence coverage (%)	MASCOT scores
N1 ~90 kDa	Transferrin binding protein A [*Pasteurella multocida*], Acc. number CAD90055	87808.22	9.71	58.12%	332
	TonB-dependent lactoferrin and transferrin receptor [*Pasteurella multocida* subsp. multocida str. P52VAC], Acc. number ZP_15715832	87808.22	9.64	58.12%	338
	omp87 protein [*Pasteurella multocida*] Acc. number CAD20126	87688.32	5.99	50.63%	181
	Protective surface antigen D15/OMP assembly factor Yae^+^ precursor Acc. number YP_005177207	87800	6.58	52.47%	178
	Hypothetical protein KCU_10206 [*Pasteurella multocida* subsp. multocida str. P52VAC] Acc. number ZP_15715705	87938.5	6.58	53.86%	185
	hmbR Acc. number CAD58169	77400	9.75	41.26%	207

N2 ~75 kDa	TonB-dependent lactoferrin and transferrin receptor [*Pasteurella multocida* subsp. multocida str. P52VAC] Acc. number ZP_15715832	87808.22	9.64	34.69%	70.3
	Transferrin binding protein A [*Pasteurella multocida*] Acc. number CAD90055	89284.06	9.71	33.08%	68.8

N3 ~50 kDa	Outer membrane protein A [*Pasteurella multocida*] Acc. number ADF95751	38433.84	9.54	78.21%	593
	Membrane protein, aromatic hydrocarbon degradation family Acc. number YP_005176018	46700	9.44	55.73%	321
	Long-chain fatty acid transport protein [*Pasteurella multocida* subsp. gallicida P1059] Acc. number ZP_18469055	47545.25	9.44	57.79%	331
	pm1069 protein [*Pasteurella multocida*] Acc. number ACT85941	47515.12	9.3	50.80%	314

N4 ~45 kDa	47 kDa outer membrane protein [*Pasteurella multocida* subsp. multocida str. 3480] Acc. number YP_006239073	47270.14	9.34	33.26%	97.7
	Hypothetical protein AAUPMB_11295, partial [*Pasteurella multocida* subsp. multocida str. Anand1_buffalo] Acc. number ZP_15708593	18875.39	8.69	62.50%	175
	Long-chain fatty acid transport protein [*Pasteurella multocida* subsp. gallicida P1059] Acc. number EJZ79884	47545.25	9.44	32.96%	97.4
	Pm 1069 protein (OMP P1 precursor) Acc. number ACT85941	47500	9.3	26.88%	90
	Glycerophosphodiester phosphodiesterase [*Pasteurella multocida*] Acc. number WP_005752028	41223	6.57	34%	82

N5 ~40 kDa	Outer membrane protein A [*Pasteurella multocida*] Acc. number ADF95751	38433.84	9.58	78.21%	593
	Hypothetical protein PM0786 [*Pasteurella multocida* subsp. multocida str. Pm70] Acc. number NP_245723	38121.61	9.44	61.19%	409
	Hypothetical protein AAUPMB_08244, partial [*Pasteurella multocida* subsp. multocida str. Anand1_buffalo]	20826	N.A.	N.A.	103

N6 ~33 kDa	Outer membrane protein H [*Pasteurella multocida*] Acc. number EJZ80794	35652.51	9.75	43.24%	250
	39 kDa adhesive protein [*Pasteurella multocida*] Acc. number ABR27206	35248.11	9.14	60.80%	434

N7 ~30 kDa	Outer membrane protein H [*Pasteurella multocida*] Acc. number EJZ80794	35652.51	9.75	51.65%	232
	Adhesive protein [*Pasteurella multocida*] Acc. number ABX58059	33740.43	9.58	37.06%	243
	39 kDa adhesive protein [*Pasteurella multoc*ida] Acc. number ABR27206	35248.11	9.14	60.80%	434

N8 ~25 kDa	Outer membrane protein H [*Pasteurella multocida*] Acc. number EJZ80794	35652.51	9.75	51.65%	232
	39 kDa adhesive protein [*Pasteurella multocida*] Acc. number ABR27206	35248.11	9.14	36.42%	208
	Hypothetical protein AAUPMB_21592, partial [*Pasteurella multocida* subsp. multocida str. Anand1_buffalo]	15131	N.A.	N.A.	66
	Hypothetical protein AAUPMB_08244, partial [*Pasteurella multocida* subsp. multocida str. Anand1_buffalo]	20826	N.A.	N.A.	103

IR1 ~125–127 kDa	HgbA [*Pasteurella multocida*] Acc. number AAQ14873	110664.92	9.04	34.91%	229
	TonB-dependent hemoglobin/transferrin/lactoferrin receptor family protein [*Pasteurella multocida* subsp. multocida str. 3480] Acc. number YP006239903	11068.5	9.15	33.23%	179
	Outer membrane receptor protein, mostly iron transport [*Pasteurella multocida* subsp. gallicida P1059] Acc. number ZP_18468195	110193.67	9.16	34.92%	229
	iroA [*Pasteurella multocida*] Acc. number CAD58140	110287.54	8.74	28.96%	194
	Hypothetical protein AAUPMB_19760, partial [*Pasteurella multocida* subsp. multocida str. Anand1_buffalo]	50170			97

IR2 95–97 kDa	Ligand-gated channel protein [*Pasteurella multocida*] Acc. number WP_005752163	95786	9.07	37%	127
	Heme acquisition system receptor [*Pasteurella multocida* subsp. multocida str. HN06] Acc. number YP_005364287	95788	9.14	35%	126
	TonB-dependent lactoferrin and transferrin receptor [*Pasteurella multocida* subsp. multocida str. P52VAC] Acc. number ZP_15715832	87808.22	9.64	48.82%	263
	Transferrin binding protein A [*Pasteurella multocida*] Acc. number CAD90055	89284.06	9.71	48.39%	266

IR3 ~75 kDa	TonB-dependent lactoferrin and transferrin receptor [*Pasteurella multocida* subsp. multocida str. P52VAC] Acc. number ZP_15715832	87808.22	9.64	54.97%	372
	Transferrin binding protein A [*Pasteurella multocida*] Acc number CAD90055	89284.06	9.71	52.25%	357
	hmbR [*Pasteurella multocida*] Acc. number CAD58169	77413.93	9.75	44.25%	318

IR4 ~50 kDa	Long-chain fatty acid transport protein [*Pasteurella multocida* subsp. gallicida P1059] Acc. No. EJZ79884	47545.25	9.44	40.63%	144
	47 kDa outer membrane protein [*Pasteurella multocida* subsp. multocida str. 3480] Acc. number YP_006239073	47270.14	9.34	41%	144
	Hypothetical protein PM1069 [*Pasteurella multocida* subsp. multocida str. Pm70] Acc. number NP_246006	47831.56	9.57	41.31%	144
	Membrane protein, aromatic hydrocarbon degradation family [*Pasteurella multocida* 36950], Acc. number YP_005176018	46776	9.04	47%	107
	Translocation protein TolB [*Pasteurella multocida*] Acc. number WP_016533064	46017	9.04	33%	67
	HexD, partial [*Pasteurella multocida*], Acc. number WP_005752881	40030	9.34	46%	89
	TolC protein [*Pasteurella multocida* subsp. multocida str. 3480] Acc. number YP_006239672	43588	9.44	34%	64
	CexD [*Pasteurella multocida*] Acc. number AAF67275	42856	9.28	43%	86
	Outer membrane protein A [*Pasteurella multocida*] Acc. number AEC04319	38459.85	9.54	82.40%	676
	Hypothetical protein PM0786 [*Pasteurella multocida* subsp. multocida str. Pm70] Acc. number NP_245723	38121.61	9.44	66.86%	459

IR5 ~40 kDa	Outer membrane protein H [*Pasteurella multocida*] Acc. number EJZ80794	33740.43	9.58	44.09%	376
	Adhesive protein [*Pasteurella multocida*] Acc. number ABX58059	33740.43	9.58	66.13%	449
	Hypothetical protein AAUPMB_21592, partial [*Pasteurella multocida *subsp. multocida str. Anand1_buffalo]	15131	N.A.	N.A.	66
	Hypothetical protein AAUPMB_08244, partial [*Pasteurella multocida* subsp. multocida str. Anand1_buffalo]	20826	N.A.	N.A.	103

IR6 ~33 kDa	39 kDa adhesive protein [*Pasteurella multocida*] Acc. number ABR27206	35248.11	9.14	57.41%	421
	Outer membrane protein A [*Pasteurella multocida*] Acc. number ADF95751	38433.84	9.54	51.12%	178
	Outer membrane protein A [*Pasteurella multocida*] Acc. number ADF95751	38433.84	9.54	51.12%	178

IR7 ~30 kDa	Outer membrane protein H [*Pasteurella multocida*] Acc. no. CBN80564	33740.43	9.58	28.12%	138

IR8 ~25 kDa	MltA-interacting MipA protein, partial [*Pasteurella multocida*], Acc. number WP_020751274	24721	9.62	55%	66
	Hypothetical protein AAUPMB_08244, partial [*Pasteurella multocida* subsp. multocida str. Anand1_buffalo]	20826	9.62	55%	95

^a^Bands correspond to Figure 1.

**Table 2:** The immunodominant bands obtained in western blot analysis by using different type of antiserum against outer membrane proteins of *Pasteurella multocida*.

Serial number	Antisera	Bands obtained	Approx. molecular weight (kDa)
1	Hyperimmune sera	5	125, 70, 38, 36, 23
2	Field sera	4	38, 36, 30, 24
3	Vaccinated sera	6	38, 36, 35, 33, 32, 23
4	Apparently normal animal sera	2	38, 36
